# Anatomical and functional outcomes of short-term DensironXTRA heavy silicone oil for rhegmatogenous retinal detachments: a comparative case series

**DOI:** 10.1038/s41598-023-30210-0

**Published:** 2023-03-06

**Authors:** Tina Felfeli, Fahmeeda Murtaza, Joshua Herman, Austin M. Pereira, Mark S. Mandelcorn, Efrem D. Mandelcorn

**Affiliations:** 1 Department of Ophthalmology and Vision Sciences, University of Toronto, Toronto, ON Canada; 2 Management and Evaluation (IHPME), Dalla Lana School of Public Health, The Institute of Health Policy, University of Toronto, Toronto, ON Canada; 3 Toronto Health Economics and Technology Assessment (THETA) Collaborative, Toronto General Hospital, Eaton Building, 10th Floor, Toronto, ON M5G 2C4 Canada; 4 Temerty Faculty of Medicine, University of Toronto, Toronto, ON Canada; 5 Department of Ophthalmology, Toronto Western Hospital, University Health Network, 6E-432, 399 Bathurst St, Toronto, ON M5T 2S8 Canada

**Keywords:** Outcomes research, Retina

## Abstract

To assess the safety and efficacy of short-term DensironXTRA tamponade for repair of complicated rhegmatogenous retinal detachments (RRD). This is a retrospective consecutive case series of patients undergoing pars plana vitrectomy (PPV) with intravitreal DensironXTRA and a comparator group with gas (sulfur hexafluoride (SF_6_) or perfluoropropane (C_3_F_8_)) tamponades by a single surgeon between January 2017 and November 2020 at a tertiary care centre. A total of 121 eyes with DensironXTRA and 81 comparator eyes with a gas tamponade were included. The DensironXTRA group had a significantly higher number of cases with inferior breaks (82% vs. 48%; *p* < 0.0001) and a history of previous PPV for RRD (64% vs. 12%; *p* < 0.0001). DensironXTRA was removed after a median period of 70 (IQR: 48.5–105.5) days. There was similar anatomical success in both the comparator gas tamponade and DensironXTRA groups (98.8% vs. 97.5%, *p* = 0.6506). Although both groups experienced a significant improvement in visual acuity, this change was significantly higher in the comparator gas tamponade group versus DensironXTRA group (*p* = 0.0017). There was no significant change in IOP in the DensironXTRA group (mean difference − 0.7; 95% CI − 1.753 to 0.331, *p* = 0.1785). The rates of complications were low and not significantly different between the two groups. There was no evidence for central macular thinning with DensironXTRA compared to the contralateral eye without RRD as well as with DensironXTRA in situ versus after its removal. DensironXTRA is a promising short-term tamponade agent with good anatomical and functional outcomes and low rates of complications for the repair of complicated RRDs.

## Introduction

Since Cibis et al.^1^ pioneered the use of silicone oil as a vitreous substitute in patients with rhegmatogenous retinal detachments (RRD) in 1962, silicone oil has been used as an endotamponade in the management of RRDs, particularly those complicated by advanced proliferative vitreoretinopathy (PVR), giant retinal tears or those secondary to trauma^2^. The Silicone Study Report 4 demonstrated significantly better anatomic and visual outcomes with conventional silicone oil versus sulfur hexafluoride (SF_6_) in patients with RRD and PVR, but a significantly greater intraocular pressure (IOP) elevation in eyes with silicone oil^3^.

Densiron (FLUORON GmbH, Neu-Ulm, Germany), one of two heavy silicone oils (HSO) approved for clinical use, is a mixture of 5000 milliPascal (mPas) silicone oil and 3.5 mPas perfluorohexyloctane (F6H8)^2^. Interim results from the HSO Study, comparing Densiron and conventional silicone oils, found no significant differences in anatomic success rates or visual outcomes in eyes with inferior RRD associated with PVR^4^. Other studies have also found similar results when comparing both oils^5,6^, with limited evidence to support the superiority of Densiron over conventional silicone oil^7^. Nevertheless, Densiron offers promising anatomic and functional outcomes in complicated RRDs^8–13^. Indications for use of heavy tamponades include patients with PVR, previous blunt and penetrating eye trauma, breaks or tears in the lower fundus periphery, giant tears, or large, multiple, and posterior breaks, RRD associated with a macular hole, a posterior staphyloma, or recurrent macular holes and patients’ inability to keep an appropriate posture postoperatively^14^. Uptake of HSOs such as Densiron has been limited by concerns regarding complications^15,16^, including emulsification^5,9,11^, glaucoma^4,6^, inflammatory reactions^11,13,17^, cataract formation^5,8,10,13,18^, IOP elevation^5,8–10,13,17–19^, and intraretinal and subretinal fibrosis^8,9^ occurring in a time-dependent manner^15^. Existing studies have also demonstrated transient macular thinning with Densiron68 tamponade in situ and recovery after removal^20^.

New generation of Densiron includes DensironXTRA, which has a lower viscosity (1200 cSt) than Densiron68, improving ease of injection with 25-gauge systems, and enhancing simplicity of removal with a lower emulsification rate^21,22^. Given the paucity of literature, it is difficult to establish the safety and efficacy of DensironXTRA in the management of complicated RRDs. Herein, we retrospectively compare the safety and efficacy of DensironXTRA as a short-term agent in the management of challenging RRDs to routine RRD cases using gas tamponades as a reference in a single vitreoretinal surgeon’s practice. We also examine the optical coherence tomography (OCT) changes in patients with DensironXTRA tamponade for RRDs.

## Methods

A retrospective review was conducted of all consecutive RRDs that underwent PPV with DensironXTRA at a single vitreoretinal surgeon’s practice (EDM) at Toronto Western Hospital, University of Toronto, Canada between January 2017 and November 2020. Both cases with primary RRD and previously failed RRD surgeries were included. Use of DensironXTRA for other indications such as perforating ocular trauma or choroidal hemorrhage was excluded. A comparator gas tamponade group of consecutive RRDs that underwent PPV with gas tamponades (sulfur hexafluoride (SF_6_) or perfluoropropane (C_3_F_8_)) between January 2020 and November 2020 were also selected. Patients’ clinical characteristics, surgical details, and outcomes (anatomical success and visual acuity) were reviewed.

Ethics approval was obtained from Institutional Review Board for Human Subjects Research at the University Health Network, University of Toronto (reference number: 21-5183), for conduction of the retrospective review and the study adhered to the tenets of the Declaration of Helsinki. Due to the nature of this retrospective study and the preserved anonymity of patients, a waiver of informed consent was obtained from University Health Network, University of Toronto.

### Surgical technique

Standard three-port PPV using the 23-gauge constellation system was utilized for all cases. A complete and thorough core and peripheral vitrectomy was performed using scleral depression and peripheral vitreous shave to the vitreous base. All pre-retinal membranes were peeled prior to fluid-air exchange draining through the peripheral break. Endolaser was applied around the retinal breaks and DensironXTRA was used to fill the vitreous cavity at the end of the case. Discovisc viscoelastic was placed in the anterior chamber in aphakic and pseudophakic cases to prevent anterior oil migration. The sclerotomies were closed with scleral needling technique as previously described^23–25^.

Within approximately 3 months post-operatively, using the Constellation Vitrectomy platform, DensironXTRA removal was performed with the Viscous Fluid Extractor as a single bubble using a 23- gauge cannula (Video 1 and supplementary file 1). Following this, residual oil droplets were aspirated with the vitreous cutter followed by a fluid-air exchange to sandwich residual oil at the air–water interface^26^. Post-operative follow-up visits were arranged on the first post-operative day, one week, one month, three months, six months and additional timepoints as needed.

### Optical coherence tomography imaging analysis

Images were captured on Zeiss CIRRUS HD-OCT 5000/500. A comprehensive set of OCT features including the presence of macro- and microstructural changes were reviewed at the baseline and last follow-up. These features included: (i) central subfield macular thickness (CSMT, defined as average macular thickness in the central 1-mm grid, automatically generated from the raster scan protocol using built-in software); (ii) macular cube volume (thickness of the tissue from the ILM to the RPE in the macular area); (iii) intraretinal fluid (IRF, defined as accumulation of fluids in retinal layers); (iv) subretinal fluid (SRF), and; (v) ERM (visually significant fibrocellular tissue affecting the central 3 mm of central macular OCT scan, Stage 1 or worse)^27^. All OCTs with poor signal quality (< 5/10) due to imaging techniques were excluded from the analysis. OCT imaging analysis measurements were retrospectively collected from the contralateral eyes without RRD of all study patients and used for the comparative analyses.

OCT data was pooled into two categories, short-term postoperative defined as < 180 days (± 15 days) following the initial PPV for RRD repair, and long term postoperative. In cases where multiple short term OCT scans were taken, the OCT closest to surgery date was selected, provided that the scan quality and signal strength were acceptable. Long-term was defined as any scan > 180 days from PPV for RRD repair, and in cases of multiple long-term scans, the furthest from surgery date was selected, provided that the scan quality and signal strength were acceptable.

### Statistical analysis

Distribution of continuous variables was examined using histograms, box-whisker plots and Kolmogorov–Smirnov tests. Numerical variables were summarized using descriptive measures using counts and percentages, means and standard deviation for normally distributed continuous variables as well as median and interquartile rage (IQR) for not normally distributed data. For within DensironXTRA analyses paired sample t-test was used. For comparisons between and the comparator gas tamponade group, the independent samples t-test was used for normally distributed continuous data and the Wilcoxon signed-rank test as a non-parametric test equivalent. For categorical data, Chi-squared test and Fisher’s exact test were used. Snellen visual acuity was converted to logarithm of the minimum angle of resolution (logMAR) values. The logMAR values for visual acuity of “counting fingers,” “hand motion,” “light perception” and “no light perception” were assigned 2, 2.3, 2.7 and 3, respectively, based on previously published literature^28^. The change in visual acuity from baseline to last follow-up was calculated and compared between the DensironXTRA and comparator gas tamponade group using the Wilcoxon signed-rank test.

A multivariable linear regression was used to determine the association of final visual acuity (continuous) with presence of DensironXTRA while adjusting for confounders. Relevant covariates were identified a priori for inclusion in the model based on clinical relevance and existing literature. The following covariates were included: age, baseline visual acuity, history of previous retinal detachment and extent of retinal detachment. Results were reported as slopes or “parameter estimates” and 95% confidence intervals (95% CI).

For the OCT imaging analysis, affected eyes were compared to contralateral eyes without RRD in both the long and short-term using two-sample t-tests. A separate analysis was performed on affected eyes with OCT scans taken during and after DensironXTRA tamponade. The latest OCT on record with DensironXTRA, was used for the “DensironXTRA in situ” group and was compared to the most recent OCT on record after DensironXTRA. These groups were compared using a paired-sample t-test.

All analyses were performed using SAS software (SAS ONDEMAND FOR ACADEMICS, 3.8 (Enterprise Edition)). A *p*-value of 0.05 was considered for statistical significance.

## Results

**Figure 1:**
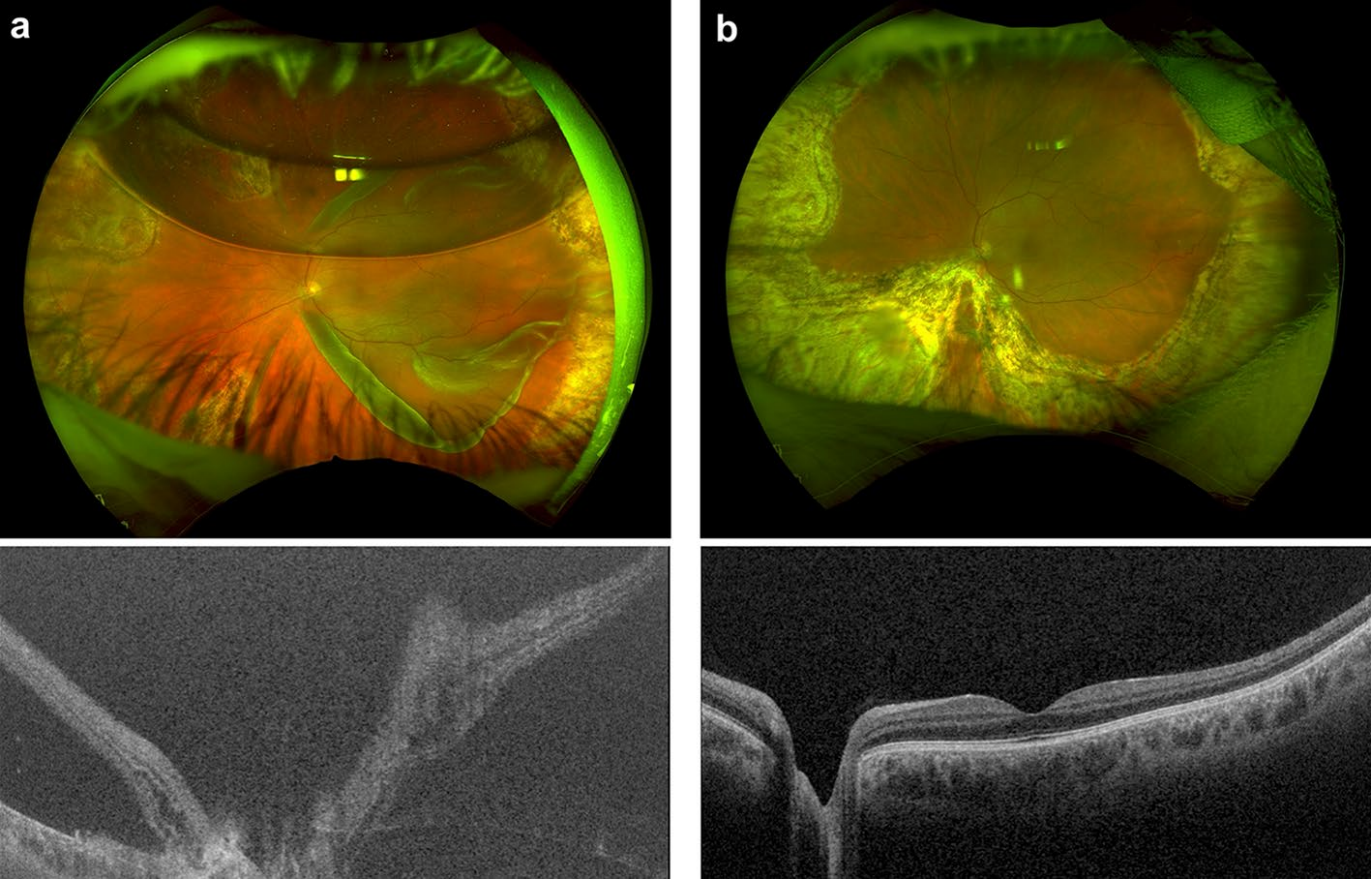
Representative case of a 61-year-old patient with a macula-off rhegmatogenous retinal detachment and giant retinal tear with gas tamponade in situ from a previously failed pars plana vitrectomy (**a**), repaired with DensironXTRA tamponade. Good anatomical outcome at 6 months post-operatively with the DensironXTRA tamponade removed (**b**).

A total of 121 eyes with DensironXTRA and 81 eyes with gas tamponade from the comparator group undergoing RRD repair were included in the analysis. The pre-operative RRD features are summarized in Table 1. The mean patient age was 63.0 (SD 14.5, range 22–90) and 62.0 (SD 12.0, range 25–91) years for the DensironXTRA and comparator gas tamponade groups, respectively. Eighty-two percent of eyes (98) in the DensironXTRA group had breaks in the inferior retina and 63.6% (77) patients had a history of previous PPV surgery for RRD (Fig. 1). Amongst the DensironXTRA cases, PVR was noted pre-operatively and post-operatively in 23.9% (29) and 2.5% (3) of eyes, respectively. None of the cases in the gas tamponade group had PVR pre or pos-operatively.

**Table 1:** Baseline characteristics of patients and eyes with DensironXTRA and gas tamponade comparator groups included in the study.

Variables	DensironXTRA Group (n = 121)	Comparator Gas Tamponade Group (n = 81)	*p* value
Age, years; mean (SD)	63.0 (14.5)	62.0 (12.0)	0.4277
Sex, female; no. (%)	41 (33.9)	18 (22.2)	0.0837
Eye, right; no. (%)	58 (47.1)	41 (50.6)	0.8019
History of previous retinal detachment; no. (%)	77 (63.6)	10 (12.4)	< .0001
History of previous retinal detachment in fellow eye; no. (%)	15 (13.9)	9 (11.3)	0.6627
Past Ocular History; no. (%)			0.0075
Age-related macular degeneration	3 (17.6)	0	
Diabetic Retinopathy	3 (17.6)	0	
Glaucoma	3 (17.6)	2 (10)	
Pre-operative pathologies; no. (%)			
Epiretinal membrane	6 (4.9)	3 (3.7)	0.7432
High Myopia	19 (15.7)	18 (22.2)	0.2682
Lattice	26 (21.5)	32 (39.5)	0.0070
Proliferative vitreoretinopathy	29 (23.9)	0	
Macular status; no. (%)			0.8331
On	35 (31.2)	22 (27.2)	
Off	10 (8.9)	7 (8.6)	
Split	67 (59.8)	52 (64.2)	
Extent of retinal detachment, clock hours; mean (SD)	6.9 (3.0)	5.5 (2.2)	0.0038
Inferior retinal detachment; no. (%)	79 (83.2)	31 (40.8)	< .0001
Presence of inferior breaks; no. (%)	98 (81.7)	38 (47.5)	< .0001
Number of breaks; mean (SD)			
Total number of breaks	2.7 (2.4)	3.1 (1.9)	0.0416
Inferior breaks	1.9 (1.5)	0.5 (0.5)	< .0001
Breaks inside retinal detachment	2.2 (2)	2.5 (1.5)	0.0168
Breaks outside retinal detachment	0.5 (1.1)	0.7 (1.1)	0.0109
Vitreous Hemorrhage; no. (%)	25 (20.7)	18 (22.2)	0.7905
Pre-operative lens status; no. (%)			0.0435
Phakic	68 (56.2)	34 (41.9)	
Pseudophakic	53 (43.8)	46 (56.8)	
Aphakic	0	1 (1.2)	
Pre-operative visual acuity, logMAR; median (IQR) / Snellen	1.3 (0.3–2.3) / 20/400	1 (0.1–2.3) / 20/200	0.2656
Visual acuity in fellow eye, logMAR; median (IQR) / Snellen	0.3 (0–3) / 20/40	0.2 (0–0.6) / 20/32	0.0005

No., number; IQR, interquartile range; SD, standard deviation; LogMAR, logarithm of the minimum angle of resolution.

DensironXTRA was removed after a median period of 70 (IQR: 48.5–105.5) days. DensironXTRA tamponade was removed in all cases except in 16 (13.2%) patients for whom it was left in situ as per patient request and/or based on poor visual potential due to pre-existing ocular pathologies (such as amblyopia or age-related macular degeneration). For eyes that were phakic at baseline, 10.2% (12) developed a visually significant cataract and 36.4% (28) had phaco-vitrectomy at the time of DensironXTRA removal. The anatomical success was not significantly different in the comparator gas tamponade group (98.8%) compared to the DensironXTRA group (97.5%, *p* = 0.6506). The median post-operative visual acuity for the DensironXTRA group at one week, 1 month, 3 months, 6 months and last follow-ups was 1.3 (IQR 0.3–2.3; 20/400), 1 (IQR 0.3–2.3; 20/200), 0.7 (IQR 0.1–2.3; 20/100), 0.7 (IQR 0–2; 20/100) and 0.7 (0–2.3; 20/100), respectively. The median change in visual acuity from pre-operative (1.3, IQR 0.3–2.3; 20/400) to post-operative visit (0.7, IQR 0–2.3; 20/100) significantly improved amongst the DensironXTRA group (mean of − 0.4911, 95% CI − 0.6574–0.3248, *p* < 0.0001). The median change in visual acuity from baseline to last follow-up was significantly higher in the comparator gas tamponade group compared to the DensironXTRA group (0.69 [IQR 0.30–1.69] versus 0.30 [IQR 0.07–1]; *p* = 0.0017). When comparing the visual acuity between the Densiron group with oil removed (excluding those with oil in situ at last follow-up) and the comparator gas tamponade group, the visual acuity was still lower for DensironXTRA group (0.7, IQR 0.3–1.3; 20/100) than the comparator gas tamponade group (0.3, IQR 0.2–0.5; 20/40; *p* < 0.0001).

Throughout the post-operative follow-ups at one week, 1 month, 3 months, 6 months and last follow-up, the mean IOP for the DensironXTRA group was 12.5 (SD 5.8), 12.6 (SD 4.2), 12.4 (SD 4.5), 12.9 (SD 3.9) and 13.1 (4.2) mmHg, respectively. There was no significant change in IOP from pre-operative (12.9, SD 3.9, range 4–23 mmHg) to the last post-operative follow-up (13.1, SD 4.2, range 1–21 mmHg) for eyes in the DensironXTRA group (mean of − 0.7; 95% CI − 1.753–0.331, *p* = 0.1785).

With regards to post-operative complications, no significant differences in rate of IOP elevation, ERM and IRF were noted between the DensironXTRA and comparator gas tamponade groups anytime throughout the follow-up periods (Table 2). The DensironXTRA group, however, had a higher proportion of eyes with persistent SRF compared to the comparator gas tamponade group (30, 25.4% vs. 9, 11.3%; *p* = 0.0174).

**Table 2:** Post-operative outcomes of eyes with DensironXTRA and gas tamponade comparator groups included in the analysis.

Variables	DensironXTRA Group (n = 121)	Comparator Gas Tamponade Group (n = 81)	*p* value
Surgical details; no. (%)			
Scleral buckle	17 (14.1)	14 (17.3)	0.5319
Phaco-vitrectomy	6 (4.9)	3 (3.7)	0.7432
Retinectomy	39 (32.2)	10 (12.5)	0.0014
Tamponade type^*^; no. (%)			< 0.0001
Sulphur hexafluoride	0	22	
Perfluoropropan	0	53	
Densiron68	121	0	
Last post-operative visual acuity, logMAR; median (IQR) / Snellen	0.7 (0–2.3) / 20/100	0.3 (0.2-0.5) / 20/40	< 0.0001
Oil in-situ	1.7 (1–2.3) / CF		0.0238
Oil Removed	0.7 (0.3–1.3) / 20/100		< 0.0001
Last post-operative IOP, mmHg; median (IQR)	13.1 (4.2)	N/A	
Last post-operative visual acuity in fellow eye, logMAR; median (IQR) / Snellen	0.2 (0–1.3) / 20/30	0.2 (0–1) / 20/30	0.7525
Last post-operative IOP in fellow eye, mmHg; median (IQR)	14.1 (3.4)	N/A	
Lens status at last follow-up; no. (%)			0.0801
Phakic	23 (20.9)	26 (32.1)	
Pseudophakic	84 (76.4)	55 (67.9)	
Aphakic	3 (2.7)	0	
Final Success; no. (%)	118 (97.5)	80 (98.8)	0.6506
Follow-up duration, days; median (IQR)	217 (1–1012)	154 (42–998)	0.8081
Post-operative complications; no. (%)			
Persistent subretinal fluid	30 (25.4)	9 (11.3)	0.0174
Epiretinal membrane	10 (8.5)	11 (13.9)	0.2456
Intraretinal fluid	6 (5.1)	6 (7.6)	0.5487
Elevated intraocular pressure (IOP, >20 mmHg)	12 (10.3)	8 (10)	1
New cataract^‡^	12 (10.2)	25 (30.9)	0.0003
Silicone oil emulsification	0	N/A	
Corneal haze	4 (3.4)	0	
Proliferative vitreoretinopathy	3 (2.5)	0	

No., number; IQR, interquartile range; SD, standard deviation; LogMAR, logarithm of the minimum angle of resolution; IOP, intraocular pressure; CF, counting fingers. N/A, not available/not applicable.

^‡^In cases that were phakic at baseline.

Given that the pre-operative characteristics of the DensironXTRA were more complex compared to the gas tamponade group, the multivariable linear regression analysis (adjusted for age, baseline visual acuity, history of previous retinal detachment and extent of retinal detachment) suggested that final visual acuity was significantly associated with presence of DensironXTRA (parameter estimate: 0.42, 95% CI 0.22–0.63, *p* < 0.0001, Table 3). Baseline visual acuity was also a significant predictor of final vision outcomes.

**Table 3:** Multivariable linear regression analysis results for visual acuity outcomes and their association with presence of DensironXTRA.

Parameter	Estimate	95% confidence limits	*p* value
DensironXTRA	0.385	0.183–0.586	< 0.0001
Age	− 0.000	− 0.006–0.006	0.4467
History of previous retinal detachment	0.051	− 0.150– 0.253	0.6342
Extent of retinal detachment	− 0.000	− 0.032–0.031	0.0950
Last post-operative visual acuity	0.249	0.141–0.357	< 0.0001

### Optical coherence tomography imaging analysis

There were 90 eyes with adequate follow up and OCT data for the imaging analysis. Measurements from affected eyes included CSMT (289.44 µm (SD 95.23)), and mean macular cube volume (10.75 µm (SD 1.65)), with 17.6%, 7.6%, and 25% having ERM, SRF and IRF, respectively. Measurements from contralateral eyes included CSMT (278.04 µm (SD 46.62)), and mean macular cube volume (10.00 µm (SD 0.75)), with 16.2%, 2.9%, 7.4% having ERM, SRF and IRF, respectively. OCT parameters including CSMT (*p* = 0.8864), and mean macular cube volume (*p* = 0.4225) were not significantly different between the DensironXTRA and contralateral eyes. The presence of IRF was significantly more common amongst DensironXTRA (25.0%) compared to contralateral eyes (7.4%; *p* = 0.005).

Amongst 33 eyes with OCTs available for DensironXTRA in-situ (median 42.58 days from PPV; IQR 26.36–86.18)) and following DensironXTRA removal (median 196.69 days from removal; IQR 60.83–544.46), no significant differences were observed in CSMT (283.2 vs. 289.15; *p* = 0.6), and mean macular cube volume (10.7 vs. 10.8; *p* = 0.57).

## Discussion

To our knowledge, this is the first comparative study looking at the efficacy and safety of DensironXTRA as a short-term agent versus gas in complicated RRDs. Overall, our findings demonstrated that cases undergoing DensironXTRA are more challenging but with good anatomical and functional outcomes post-operatively. The goal of this study was to investigate the safety profile of DensironXTRA in comparison to gas tamponades as a reference standard. Our findings demonstrated a low rate of complications for DensironXTRA in comparison to the comparator gas tamponade group, which consisted of less challenging RRDs.

In our study, anatomic success was slightly greater in the comparator gas tamponade group (98.8%) than the DensironXTRA group (97.5%), however, a significantly greater number of eyes in the DensironXTRA group had a prior history of RRD, inferior retinal detachment and inferior breaks. As such, the differences seen in the DensironXTRA and comparator gas tamponade group success rates may be largely driven by differences in complexity of the cases. Our anatomic success rates for the DensironXTRA group were comparable or higher than those reported for Densiron in the existing literature^8–10,17–19,29–31^.

We observed a significant improvement in final visual acuity from baseline in the DensironXTRA group. Our multivariable linear regression analysis suggested that baseline visual acuity was a significant predictor of final vision outcomes. Although the preoperative visual acuity was not significantly different between the two groups, the postoperative visual acuity was significantly better in the comparator gas tamponade group than DensironXTRA group. This may be a result of the significantly higher history of previous RRD, larger extent of detached retina and greater likelihood of persistent subretinal fluid in the DensironXTRA group post-operatively. The difference in final visual outcomes between DensironXTRA and comparator gas tamponade groups was noted despite the lower rates of ERM and cataract in the DensironXTRA group compared to the gas tamponade group. Other studies on Densiron68 as an intraocular tamponade for RRDs have noted good visual acuity recovery post-operatively for primary RRDs^29^. Amongst eyes with previously failed complicated retinal detachments, Herbrig et al., did not find significant changes in preoperative to postoperative visual acuity^17^. Similar to our experience, several other studies have found notable improvement in visual acuity for complicated RRDs with proliferative vitreoretinopathy, posterior or inferior retinal breaks^9,11,13,32^.

Based on our analysis, the complication rate in the DensironXTRA group was low, and not significantly greater than the comparator gas tamponade group. Notably, the rate of visually significant cataract formation was greater in the comparator gas tamponade group (30.9% vs. 10.2%), despite having a significantly fewer phakic eyes preoperatively as compared to the DensironXTRA group. Most importantly, no significant differences in rate of IOP elevation, ERM and IRF between the DensironXTRA and comparator gas tamponade groups were identified. Previous studies evaluating complications rates between Densiron68 and 1,000 centistoke silicone oil found cataract, raised IOP, inflammatory reaction, macular ERM, and emulsification in both groups, but these were not significantly different between the two agents^5^. One of the potential advantages of DensironXTRA compared to Densrion68 is the lower emulsification rate, as DensironXTRA contains high molecular weight components that are harder to breakup under shear stress^21,22^. In keeping with this, we found no oil emulsification amongst any of the cases included in our analysis with DensironXTRA as a short-term tamponade, which is lower than previously reported rates with Densiron68 (18.5–33%)^33–35^.

Our OCT imaging analysis showed that there is no evidence for central macular thinning with DensironXTRA compared to the contralateral eye without RRD as well as with DensironXTRA in situ versus after its removal. A meta-analysis conducted by Ghanabri et al. suggested that retinal thinning occurs with silicone oil tamponade and that silicone oil is associated with a greater degree of thinning compared to gas tamponades^36^. There is conflicting evidence whether visual acuity is affected by retinal thinning^36–39^. Other studies comparing affected eyes to contralateral eyes after silicon removal, did not demonstrate significant post-removal CMT thinning with and without the consideration of axial length^40–44^. We have also previously shown that there may be transient macular thinning with Densiron68, which recovers after tamponade removal^19^. Similarly, OCT analysis of parafoveal ganglion cell and inner plexiform layer have shown transient thinning with silicone oil tamponade, with recovery after 6 months^37^.

It is important to note that given the retrospective nature of this study, there was an unequal complexity of cases between the DensironXTRA and the comparator gas tamponade group. Since it is not feasible and ethical to subject patients to surgical management strategies which are not optimal for their care, statistical adjustments were made for the complexity of the cases. Although the use of the multivariable regression analysis enabled us to account for variations in subject variability which could confound the study outcomes, the heavy silicone oil group may have been superior to the gas had similarly complex cases been compared. All cases were performed by one surgeon, which increases internal reliability of the findings in terms of the thoroughness of surgical techniques and complex maneuvers but may decrease the generalizability. The variability in follow-up time among patients is a limitation of the study. The visual acuity measures may have also been confounded postoperatively by progression of cataract in phakic patients. A number of the contralateral eyes used as non-RRD comparators for the OCT analysis had vitreous interface anomaly resulting in ERM and other pathologies. There was no standardized OCT imaging protocol in place throughout patient follow-ups as part of standard of care. As such, short term and long-term follow-up groups were created for better comparability of the cases. Note that long term follow-up data was not as readily available as short-term data, which only allowed us to perform long-term analysis on a smaller portion of our cases.

## Conclusions

The findings from our study suggest that anatomical and functional outcomes as well as minimal complication rates of RRD repair with DensironXTRA as a tamponade are very promising. DensironXTRA serves as an effective and safe short-term tamponade choice in the management of complicated RRDs.

## Supplementary Information

Supplementary Information.

Supplementary Video 1.

## Data Availability

The datasets generated and/or analysed during the current study are not publicly available due to risk of releasing identifiable information. Limited aspects of the data may be available from the corresponding author on reasonable request.
