# Supplementary material for: Anatomical and functional outcomes of short-term DensironXTRA heavy silicone oil for rhegmatogenous retinal detachments: a comparative case series
Source: Sci Rep. 2023 Mar 6;13:3729. doi: 10.1038/s41598-023-30210-0 (PMC9988839; doi:10.1038/s41598-023-30210-0)
Supplement: Supplementary file 1 — Supplementary Information. [file 41598_2023_30210_MOESM1_ESM.docx]

**Supplementary File 1.** This video illustrates the use of DensironXTRA heavy silicone oil for rhegmatogenous retinal detachments.
